# The Influence of Internal Marketing and Job Satisfaction on Task Performance and Counterproductive Work Behavior in an Emerging Market during the COVID-19 Pandemic

**DOI:** 10.3390/ijerph18073670

**Published:** 2021-04-01

**Authors:** Marcela-Sefora Nemteanu, Dan-Cristian Dabija

**Affiliations:** Department of Marketing, Faculty of Economics and Business Administration, Babeș-Bolyai University, 400591 Cluj-Napoca, Romania; sefora.sana@gmail.com

**Keywords:** internal marketing, job satisfaction, job performance, task performance, counterproductive work behavior, COVID-19

## Abstract

To reduce the spread of the virus, authorities have imposed restrictive measures, such as limiting movement of individuals, shutting down non-essential stores, imposing a general or local quarantine, along with physical distancing and isolation of vulnerable people. Remote working has become the ‘new normal’ for many organizations, engendering further challenges for employees, who have started experiencing anxiety, technostress caused by digitalization and lack of social interaction, frustration, occupational burden, counterproductive work behavior, exhaustion, burnout, depersonalization, and increased turnover intention. All these factors, corroborated by prolonged restrictions, have contributed to a decrease in employee satisfaction, diminishing performance and generating a counterproductive behavior. Based on Social Exchange Theory, this research plans to investigate the influence of internal marketing on job satisfaction, task performance, and counterproductive work behavior in the context of the COVID-19 pandemic in an emerging market, namely Romania. Based on a quantitative research study among 850 employees, we show that internal marketing strongly and significantly impacts job satisfaction, while insignificantly impacting task performance and counterproductive work behavior. Job satisfaction actuates task performance in a significant and positive manner, contributing to a reduction in counterproductive work behaviors. This paper highlights the effects of internal marketing orientation on job satisfaction, and the effects of job satisfaction on job performance and counterproductive work behaviors.

## 1. Introduction

The COVID-19 global pandemic has become a reality in managing the processes of organizational health, and in the recent management of human resources [1,2,3], with direct implications on employee satisfaction and performance [4], together with the effects and vast repercussions on employee wellbeing. Managers [5] and authorities [6] are not always fully aware of these effects. To remain in the market, organizations from various industries have devoted considerable effort to adapt to the turbulent conditions in their dynamics, and to identify solutions and proper strategies [6,7].

For well over a year, the new socio-economic context, especially in sanitation [8,9,10,11], has affected the way employees perform their tasks and duties. The spread of the COVID-19 pandemic [12,13,14] has demanded decision-making in terms of isolation and physical distancing of individuals, and employees in particular [15]. The restrictive measures have imposed a reorganization of internal processes and procedures regarding subsequent economic activities [16]. If at first employees’ work was suspended for various time periods [17,18], thereafter, teleworking has become the new reality [1,2,19,20]. In the context of the COVID-19 pandemic, human resources managers are facing challenges in maintaining a proper level of job satisfaction among employees, in coordinating them, and in encouraging efficiency in the performance and implementation of assumed tasks [4]. Therefore, internal marketing assumes a major role in maintaining and increasing employees’ job satisfaction [21,22], boosting organizational engagement [23,24], and increasing performance under unfamiliar conditions in the performance of tasks and duties according to job descriptions [23].

This paper plans to fill a research gap regarding the extent to which, in the context of the pandemic, internal marketing may provide a solution to increase employee satisfaction, to diminish counterproductive work behavior, and to increase task performance within an organization. Based on the Social Exchange Theory, the authors have implemented a quantitative study in an emerging market heavily impacted by the COVID-19 pandemic [13], identifying the degree to which the internal marketing orientation of organizations influences job satisfaction, the accomplishment and fulfilment of tasks, and the diminishing of unproductive work behavior among employees.

The paper is structured as follows: the following section is comprised of a literature review regarding the approach of internal marketing within organizations and its role in managing human resources in the context of the COVID-19 pandemic. Within this framework, the role of employees’ job satisfaction and performance in accomplishing tasks with regards to organizational health and stability are highlighted. The third section covers the context and research methodology, the conceptual framework, and analysis process, followed by the discussion and results in comparison with existing data from the literature. The final section consists of the theoretical contributions of the paper to the advancement of Social Exchange Theory, the managerial implications, and the limitations and research prospects.

## 2. Literature Review and Conceptual Model Development

The socio-economic crisis engendered by the COVID-19 pandemic has imposed a change in the operating strategy of all organizations, and a re-thinking of activities performed by employees [1,2,18]: the wearing of masks, constant disinfecting [25], switching from working in an office to working remotely—namely teleworking, etc. These changes have led to the use of multiple technologies and digital tools [26] to perform ordinary tasks: attending and/or organizing meetings through video conferencing, collaborating with team-mates regarding task fulfillment, preparing reports and/or presentations, using electronic registers, etc. [27]. Teleworking is not a new phenomenon and has been used to a certain extent in the past [28]. However, it has now become the preferred means of work for many organizations, especially in the service industries, for they must abide by the social distancing norms [20] and have had to reduce employee traffic [2]. This has been strongly favored by recent developments in digital technologies [29] and virtual communication [17,30]. Teleworking has been met by employers and employees with understanding and acceptance, as it is deemed an essential lever for the continuation of organizational activities in close-to-normal conditions [1,31]. Nonetheless, teleworking poses great challenges due to the lack of face-to-face social interaction [20,30], stress [3,32], and employee exhaustion [33], affecting their mental health [33,34]. During the COVID-19 pandemic, organizations must maintain an acceptable level of job satisfaction [35], increasing employee productivity [36]. In this new working environment, it is hence important for human resource management to adopt procedures and work policies capable of maintaining employee motivation [37].

The complexities of the phenomena and social implications generated by the COVID-19 pandemic—namely stress, exhaustion, anxiety, burnout, counterproductive work behaviors, along with motivation, performance, health, wellbeing, development, and employee satisfaction—can be explained with the help of the Social Exchange Theory [38]. Social exchange is founded on specific conditions, such as interdependence, social relations, and obligations between parties [39]. One of the Social Exchange Theory assumptions is that within organizational relationships, the exchange of two parties is based on reciprocity [40,41]: when employees feel supported and rewarded, they manifest positive and proactive attitudes and behaviors regarding their work, thus supporting desirable practices [42]. Essentially, the employee-employer relationship takes shape and is strengthened based on these conditions. In an organizational context, employees tend to understand employee-employer reciprocity, and are prone to manifest positive attitudes and obtain results for the benefit of the organization when the latter helps and/or supports them [38]. In internal marketing research, Social Exchange Theory shows a theoretical worth in evaluating the exchange between organization management that offers support through communication, development and rewards, and employees’ performance [43,44,45,46,47].

Such an organizational endeavor contributes not only to the physical wellbeing and mental health of employees, but also to a balancing of the climate within the organization. Under conditions of organizational stress, along with the technostress caused by excessive use of modern communication technologies, and due to teleworking, employees can feel support through better internal communication with their employer [42]. Internal communication as a component of internal marketing [21] has developed a new edge and an increased importance. Moreover, well-designed, and consistent internal marketing may contribute hugely to the reversal of the damaging effects engendered by the COVID-19 pandemic [48].

Social changes and behaviors generated by this pandemic are perceived differently by employees—to some people they seem beneficial, allowing them to spend more time with their families and to easily manage their household activities [30], but to other people, the exposure to online communication technology, increased mobile device usage, learning of new procedures and the necessity of online platforms combined with the lack of social interactions and limiting of social gatherings has fostered discomfort, generating anxiety, stress, and exhaustion [1,20,34].

### 2.1. Internal Marketing

Internal marketing represents the human resource management of an organization, wherein the employee is considered its internal client [49]. This approach was considered a possible solution to the delivery of high-quality services by satisfying employee needs in the 1970s [50]. According to this philosophy [51], employees pose as internal clients, whose jobs are assimilated with the internal products of the organization. These must be comprised of tasks and duties that satisfy the needs of the internal clients. In this manner, the organization, along with its internal clients, can achieve their set objectives and strategies. Thereafter, this concept was developed, experiencing three stages of evolution [52]: the first one was aimed at employee satisfaction and motivation [51,53]; the second was aimed towards the consumer (external client), internal marketing becoming a form of integration of various vital functions enhancing customer relations [54]; whereas the third stage transformed internal marketing in a mechanism for the implementation of organizational strategies [55].

Internal marketing has been approached oftentimes in the organizational context [23], having been analyzed through communication of the organization’s vision, and the development and rewarding of employees [56]. Internal communication with employees is deemed the relational component of internal marketing, bearing a significant influence on the increase in job satisfaction and organizational engagement [57]. Through internal marketing, an organization ensures that the promise of a satisfying result for clients can be met with success [55], fostering positive implications in boosting employee satisfaction [21,22], subsequent organizational engagement [24,57], and in improving performance of the provided service [58]. Internal marketing orientation on an organizational level has been studied in all sectors: in the productive sector [59], in the tertiary sector [24,55,58,60], in public institutions [61], and in nonprofit organizations [29,62,63]. In stressful situations, especially the technostress engendered by the necessity or mandatory use of technological devices [64], internal marketing orientation constitutes a mode propitious for increasing performance and employee satisfaction, along with the reduction of subsequent negative results [24,58,60,65].

In internal marketing theory, there are various dimensions to this construct. One of the largely accepted models of internal marketing is comprised of the following dimensions: communicating vision, and the development and rewarding of personnel [56,66,67]. From an internal marketing perspective, communicating vision is defined as how an organization conveys its purpose, objectives, and strategies to every employee [56,68,69]. Organizations that constantly communicate their objectives and vision to their employees manage to mobilize them towards increased performance, boosting their satisfaction owing to the sense of belonging to a successful organization, along with an increased engagement towards that organization [69]. Developing personnel is a central element of internal marketing orientation [56]. It consists of all modes through which an organization facilitates the professional development of its employees, via training, materials, courses, supervisors, etc. [29,63,67]. Developing personnel as a dimension of internal marketing significantly increases job satisfaction and individual performance [70,71]. However, the impact of internal marketing on counterproductive work behaviors has been studied too little; studies in human resources show that the development of personnel contributes to a reduction in counterproductive behaviors [72].

Rewarding personnel depending on the evaluation of performance results is another important dimension in internal marketing [56,67,70]. This shows the degree of implementation of certain evaluation systems in terms of performance, and various forms of results rewards that contribute to the welfare of the organization [52,73]. Those organizations which employ performance evaluation and reward systems record better results and more highly satisfied employees [71]. When a reward system is unfair, and the social exchange between an organization and its employees is not equitable, the employees’ wellbeing and health may be negatively affected [74], leading to repercussions in terms of task management and organizational results.

### 2.2. Job Satisfaction

Job satisfaction represents a positive attitude [75,76] or favorable emotion of the employee towards the activity carried out [77], enhancing the working environment and thus contributing to a favorable atmosphere in the workplace [78]. This attitude reflects a high degree of employee wellbeing and is often associated with the desire to show more dedication towards the organization [79]. A satisfied employee will show reduced turnover intention [80,81], making considerable efforts not only at better integration, but also to represent the organization with dignity and pride [82]. Job satisfaction is perceived as a good indicator of employee wellbeing [83] or as a dimension of employee happiness regarding the workplace [84]. This employee attitude [76] is of paramount importance for human resource managers who understand that maintaining employees in the long run, and increasing engagement and performance depends on job satisfaction [79,81,85].

Job satisfaction is a complex construct [80] with multiple facets, valences, and implications. Satisfaction is assimilated with an employee’s contentment with the organization [86]; it is also an accumulation of work situations shaped by the relationship between the employee and co-workers, the relationship with supervisors, the working environment [87], value of work [88], pay grade, the manner in which the work done favors personal health, work acknowledgement, promotion opportunities, job security, and degree of organizational concern for the employee’s needs, etc. [81].

In situations that require high resilience to stress [89,90], or where employees are forced to adapt to changes in activity in their workplace [76], maintaining a high level of job satisfaction is an unrealistic goal [76]. Therefore, internal marketing is even more important for positive and sustained job satisfaction [22,24,60]. Organizations that deem their employees to be internal clients manage to maintain and, over time, increase their level of satisfaction [24]. When an organization offers sustained support to their employees in their development [91], by appreciating them, supporting them, and acknowledging their merits, they will be significantly more satisfied with their work done [60]. Personnel satisfaction is directly influenced by psychosocial support in their career and by the quality of the mentoring, which has direct implications on employee development [92]. There are close links between job satisfaction and the dimensions of internal marketing—internal communication of organizational vision, personnel development, and performance-based rewards [65,91,92,93,94]. Therefore, we postulate that:

**Hypothesis** **1** **(H1).** *Internal marketing influences job satisfaction.*

### 2.3. Individual Job Performance—Task Performance and Counterproductive Work Behavior

Job performance consists of all the completed tasks in a period by the employees of an organization, and is the total value expected by the organization from the individual behaviors of their employees [95]. When employees display positive emotions regarding their organization, they will manifest behaviors of organizational citizenship, identifying to a certain extent with it and thus increasing their productivity and the desire to complete their tasks. However, in the case of negative emotions towards the workplace, employees will display counterproductive behaviors [96].

An employee’s individual performance is touched on from the perspective of three dimensions, namely task performance, contextual performance, and counterproductive work behavior [97,98]. This approach has been highly debated in the literature [90,99,100], studies offering a multidimensional perspective on the way employees perceive their own performance or display counterproductive behaviors [97,98]. As intriguing as the analysis of counterproductive work behavior regarding employees’ individual performance may seem, its consideration is entirely justified because, if performance expresses efficiency, then counterproductivity expresses the opposite, namely employees’ inefficiency to handle the tasks and/or the duties, namely the workload. Of course, an employee’s counterproductivity may be a result of individual shortcomings, such as the lack of certain abilities, knowledge, or skills. Such behavior may be detrimental to the organization, engendering damaging effects to its organizational health [95].

Employee’s task fulfillment performance is of paramount importance to human resource management within an organization because it is linked to the efficiency of the entire activity [100]. Fundamentally, it is an accumulation of employee results, such as proper task planning so that tasks are completed on time, orientation towards result maximization and effort minimization, prioritization of important tasks over less important ones, and their efficient completion with minimal time and effort [97,98].

Counterproductive work behavior may be manifested by employees through various attitudes or actions with a negative impact [95], namely complaining to co-workers or people outside the organization about issues encountered, stress, or lack of acknowledgement in the workplace. Granted, they may exaggerate the difficulty or scale of a given task, focusing mostly on the negative aspects of tasks, and minimizing the positive ones [98]. Job performance is determined by a multitude of factors, two of which are internal marketing [23,101,102] and employee job satisfaction [24,103]. Internal communication and employee development through training are two components of internal marketing and can generate an increase in job satisfaction and employee performance [91]. Therefore, we consider that:

**Hypothesis** **2** **(H2).** *Internal marketing influences task performance.*

Internal marketing is dealt with in relation to job performance [104] and organizational performance [102], highlighting its role in creating the necessary premises for the reduction of counterproductive work behaviors of employees. Internal marketing has direct implications for the orientation and development of employees so that they are more likely to contribute to increase in client satisfaction and to enhancing organizational performance [68]. At the same time, it has been proven that internal marketing has a significant impact in diminishing unwanted organizational influences, such as turnover intention [105]. Therefore, we propose the following hypothesis:

**Hypothesis** **3** **(H3).** *Internal marketing influences counterproductive work behavior.*

Employee job performance constitutes the main vector of organizational efficiency, so human resource managers should prioritize its enhancement [95]. Job satisfaction constitutes perhaps the main driving force behind the increase in employee performance [90], thus strongly contributing to the reduction of counterproductive work behavior [106]. An increase in job satisfaction among employees will motivate them to obtain better results, to plan their work more thoroughly, and to become more efficient in task fulfillment. At the same time, they will be more careful when presenting their organization and/or their superiors to third parties; they are more likely to avoid talking down their own organization, or displaying any infamous behaviors [98], while becoming more devoted to their workload [90].

Job satisfaction plays an essential role in overcoming difficult moments, enhancing employee resilience in their organizational endeavors, even when subject to various crises, such as socio-economic, political, etc. disasters [90]. When employees are satisfied with their jobs, they perform work of a higher quality, thus contributing through advice, counselling, and recommendations to satisfying clients, and implicitly to their own satisfaction towards the organization and its endeavors [24,103], and become more adept at task fulfillment [24,91]. Individual job performance consists of a set of activities which contribute to the results of an organization. When employee needs concerning development and training, autonomy and social support are met, they will be more motivated to invest their physical, mental, and emotional energy in their work, boosting performance [107]. Therefore, we consider that:

**Hypothesis** **4** **(H4).** *Job satisfaction influences task performance.*

Negative behaviors in the workplace have been carefully approached in the literature due to the significant psychological, social, and economic implications on the working environment [108]. Previous studies highlight the negative association of job satisfaction with counterproductive work behaviors [108,109,110,111]. If work-related stress generates a counterproductive work behavior, quality relationships between employees and an atmosphere without tension favors their job satisfaction [112,113]. Among the counterproductive behaviors encountered in organizations, such as sabotage, diversion or slowdown in production, harmful behavior towards other employees, or co-workers’ verbal abuse [110], these are often the result of unsatisfactory work [114]. Therefore, we propose the hypothesis:

**Hypothesis** **5** **(H5).** *Job satisfaction influences counterproductive work behavior.*

**Figure 1:**
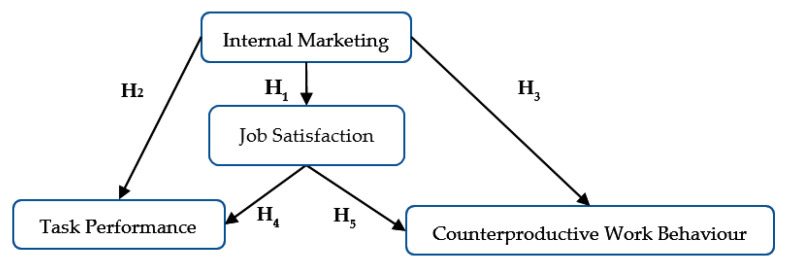
The influence of internal marketing on job satisfaction, task performance, and counterproductive work behavior.

Taking into consideration the positive influence of internal marketing and job satisfaction on task performance [24,58,60], but also with regards to the reduction of employee counterproductive work behaviors [111], we propose an analysis of the contribution of internal marketing on job satisfaction, task performance, and counterproductive work behaviors in the context of the COVID-19 pandemic in an emerging market (Figure 1).

## 3. Research Methodology

### 3.1. Research Design and Context

With a view to identifying the extent to which internal marketing influences job satisfaction, task performance and counterproductive behavior in the context of the sanitation pandemic generated by COVID-19, the authors resorted to implementing an explorative quantitative study among employees in Romania. Choosing Romania as the research context was justified because employee performance has been affected by the new pandemic context. Employees spend more time at work and report lower individual performance than before the pandemic [115,116]. This research was based on the investigation model, using self-administered online questionnaires as a tool. The invitation to participate was disseminated by the authors on different social media platforms (Facebook, LinkedIn, and Twitter), educational platforms, and directly to various organizations.

The questionnaire was filled out by Romanian residents with an employment contract valid at the time of response. The data were collected during the state of emergency and lockdown, over spring-summer of 2020, totaling 850 complete and valid questionnaires according to literature specifications [117]. Although we collected almost 1000 questionnaires, we dropped from the final sample those with missing data. Sampling was one of convenience, aiming to maintain sampling according to age and gender in accordance with the distribution specified in the Statistical Yearbook of Romania [118]. The structure of the sampling according to the type of employing organizations is presented in Table 1.

The respondents were employees residing in Romania with employment contracts at foreign private companies (31.64%), privately owned companies (50.47%), and public institutions (17.88%). The largest number of respondents were from the higher education sector (74.35%), of which most employees worked in privately owned companies (32.52%). The research was implemented within organizations with over 500 employees—11.29% were respondents of foreign private companies, 5.8% worked in privately owned companies, and 2.35% in public institutions—and within medium-sized companies ranging from 51 to 500 employees—11.64% were respondents of foreign private companies, 10.58% worked in privately owned companies, and 7.17% in public institutions—and small-sized companies ranging from 1 to 50 employees—7.6% were respondents in foreign private companies, 32.33% worked in privately owned companies, and 6.47 were respondents in public institutions. In Romania, the COVID-19 pandemic generated an emergency state in March-May 2020, when most public institutions and private companies shifted to teleworking: 60.23% of respondents in our study had to do their work through teleworking. During this time, 26.58% of our respondents reported that their job security was directly affected, while 19.76% declared they felt job insecurity. Among the respondents, 16.82% declared that they felt frustrated by having to work remotely, given the COVID-19 pandemic.

### 3.2. Variable Measurement and Data Analysis

The investigated dimensions presented in Figure 1 have been operationalized according to literature specifications, the authors using the scale of internal marketing orientation [56], job satisfaction [119], and individual work performance (Individual Work Performance Questionnaire: IWPQ) for employee task performance and counterproductive work behavior [98]. For data validity, reliability, and internal consistency, the following were employed: Cronbach α (>0.7) coefficient, KMO criterion (>0.7), Bartlett test of sphericity, and exploratory factor analysis [120,121,122]. The data are presented in Table 2. The obtained values for the Fit indicators are higher than the minimum threshold, which indicates the validity and reliability of data and allows for subsequent data analysis. The results of the factor analysis show that the considered constructs can be delimitated properly one from another and that they are stable [123].

Thereafter, all the items were included in a single factor analysis [120,121,122], which confirmed the stability of each construct [117] (KMO: 0.956 > 0.7, χ^2^: 18.058,967 ****; df: 595), the results thus highlighting five factors (Table 3). Internal marketing constitutes a dimension [56], and 3 items of the scales were removed because they loaded more factors, which indicates an inconsistency [117].

Following the analysis of the exploratory factor analysis, the research model has been adapted according to the results (Figure 1), and the aggregated data were analyzed with the help of structural equations modelling done in AMOS [70].

## 4. Results and Discussions

During structural equations modelling (SEM), the goodness-of-fit indices exceed the minimum values of sample adequacy specified in the literature: GFI, AGFI, NFI, CFI, TLI > 0.8; RMSEA, SRMR ≤ 0.08 [120,121,122], which allowed for the validation of the model (Figure 1) and data interpretation (Table 4).

The results of the SEM analysis of the sample (850 cases) highlights that the dimensions of internal marketing influence job satisfaction directly (0.721 ***) and significantly. Increasing satisfaction is directly and strongly determined by the internal marketing components. Therefore, H1 is validated. This result is corroborated with the literature, which demonstrates that the orientation of an organization towards meeting the needs of its employees [49,55] is associated with wellbeing and job satisfaction [22,24,60]. Internal marketing had no significant impact on the job performance of employees during the COVID-19 pandemic, which invalidates the H2 hypothesis. The results are rather surprising, as they contradict previous studies [68], which highlight a significant and positive link between internal marketing dimensions and job performance. Most likely the COVID-19 pandemic has baffled previous value systems and employee perceptions due to uncertainty and the return to the office being constantly postponed, teleworking thus becoming ‘the new normal’.

Internal marketing has no significant influence on counterproductive behavior, which leads to the rejection of H3. These results are also surprising, for previous studies confirmed the significant influence of internal marketing [24,58,60,68] at the organizational level [105]. Most likely the research context generated by the COVID-19 pandemic has significantly changed the perception of respondents. Nevertheless, job satisfaction has a significant and positive, though less intense impact on task performance (0.296 **), which allows for the validation of H4. This result confirms previous studies from the literature [90]. Job satisfaction has a significant and negative impact, though less intense, on counterproductive work behaviors (−0.275 **), which allows for the validation of H5. This result confirms the conclusions found in the literature [109,110,111,112].

The positive influence of internal marketing on job satisfaction was emphasized within various types of organizations. In private companies [24,71] and public institutions [124], internal marketing practices generate an increase in employee satisfaction. For instance, the implementation of internal marketing led to positive results among 355 employees within the hospitality industry, thus marking its significant and positive impact on job satisfaction [5]. Similar results were obtained from research involving Greek hospitals [124]. Especially in the context of the COVID-19 pandemic, the implementation of internal marketing is considered important for employee management [48] and to provide high-quality services [125].

The strongest evidence concerning the influence of internal marketing on job performance comes from the services sector [23,102]. A study among 617 Chinese employees in IT highlights the influence of internal marketing on psychological empowerment, a dimension which determines job performance [101]. By mediating organizational engagement, internal marketing influences job performance, an aspect also confirmed by Varshney and Varshney [90].

While little interest has been shown in the literature on studying the influence of internal marketing on counterproductive work behaviors, this has assumed a key role in the context of the COVID-19 pandemic. The reduction of counterproductive work behaviors and maintaining of a high level of job satisfaction among employees subject to various changes such as teleworking [2,20], or the introduction of new social distancing norms [20] can be achieved with the aid of internal marketing. Therefore, job satisfaction assumes the role of mediator between various precursor variables and counterproductive work behavior, diminishing its manifestation among employees [109,111,112].

## 5. Conclusions

In the context of the COVID-19 pandemic, human resource management is subject to new challenges in maintaining a high level of job satisfaction, task performance, and a reduction of counterproductive work behaviors. Internal marketing represents the way an organization may meet the needs of its employees, thus contributing to an increase in performance, and positively impacting the desired results. At the same time, internal marketing contributes to the reduction of unwanted behaviors, attitudes, and negative effects of work. This paper expands the research from internal marketing theory, thus confirming the strong and significant link between the internal marketing orientation of an organization and job satisfaction, whilst also showing that internal marketing does not directly contribute to the reduction of counterproductive work behaviors. Nevertheless, there is an indirect role of internal marketing in reducing counterproductive work behaviors by increasing job satisfaction, an attitude which has direct and significant implications for organizations, in the improvement of task performance, and the reduction of counterproductive work behavior. This paper contributes to the Social Exchange Theory [126], based on the premise that employees will foster positive attitudes towards the organization and engage with its endeavors provided they feel supported by it. Therefore, the influence of internal marketing on job satisfaction confirms the advantageous social exchange for both parties—job satisfaction has positive implications on task performance and is a determinant in the reduction of counterproductive work behaviors.

The managerial contributions of this paper consist in pointing out the importance of internal marketing in enhancing job satisfaction within an organization in the context of the COVID-19 pandemic. This crisis has come with changes in the way employees perform their activities, which have been met with resistance and a degree of concern from many employees. Maintaining job satisfaction at a high level is directly and strongly determined by the manner in which an organization communicates its vision, employee development endeavors through training, and the promotion of reward systems based on measuring employee performance. Therefore, the policy of human resources requires an approach through which employees’ needs are a central objective in maintaining a high level of job satisfaction. The obtained results reflect that internal marketing has no direct influence on the reduction of counterproductive work behaviors, but by boosting job satisfaction, there is a significant reduction in counterproductive work behaviors. The dimension of rewarding performance highlights—according to the results—a negative impact, though less intense, on job performance in the context of the COVID-19 pandemic. Increasing satisfaction comes with a desired effect of encouraging individual job performance, achieved through proper planning of tasks, their successful fulfillment, orientation towards results, and proper prioritization.

Research limitations envisage internal marketing analysis in the context of the COVID-19 pandemic only through the lens of organizational results. The context of the COVID-19 pandemic has imposed major changes in the way employees perform their work, such as massive implementation of teleworking, even by organizations and institutions that have never previously employed such a practice. We did not analyze the impact of COVID 19 on work behavior. This would be another research context and the topic of another paper. Digitalization forced by the necessity of physical distancing, the stress concerning infection, technostress, and other factors have contributed to the diminishing of employee comfort. In the future, studies will have to take into consideration the impact of teleworking on employee task performance, their work engagement and/or the decrease in turnover intention or employee intention to quit. At the same time, future studies might take into consideration the effects of stress generated by teleworking and digitalization. Human resource management is subject to new challenges for which pertinent and proper solutions must be implemented so that there is an increase in employee job satisfaction and a decrease in counterproductive work behaviors. Future research might consider applying a hierarchical linear modelling for such a research question, which would require collecting a new set of data. In a future study, organizational variables, and personal variables, for example, organizational commitment and a psychological variable, could be included as intermediary or regulating variables, and corresponding control variables could be incorporated to exclude relevant influencing factors to obtain more representative conclusions.

## Figures and Tables

**Table 1:** The structure of the sampling according to the type of employing organizations (*n* = 850).

	Foreign Private Companies		Privately Owned Companies		Public Institutions		Total	
	*n*	%	*n*	%	*n*	%	*n*	%
Gender								
Male	99	11.64	156	18.35	30	3.52	285	33.52
Female	170	20.00	273	32.11	122	14.35	565	66.47
Age								
Ages 18–25	109	12.82	151	17.76	34	4.00	294	34.58
Ages 26–40	110	12.94	151	17.76	43	5.05	304	35.76
Age 41 and over	40	5.88	127	14.94	85	10.00	252	29.64
Level of education								
High school	57	6.7	103	12.11	10	1.17	170	20.00
Professional	11	1.2	24	2.8	13	1.52	48	5.64
Higher education	201	23.64	302	35.52	129	15.17	632	74.35
Number of employees in the organization								
Between 1–50	65	7.6	275	32.35	55	6.47	395	46.47
Between 51–500	99	11.64	90	10.58	61	7.17	250	29.41
Over 500	105	12.35	64	7.52	36	4.23	205	19.52
Total	269	31.64	429	50.47	152	17.88	850	100
Number of employees affected by COVID-19 in emergency state period								
Felt job insecurity	110	12.94	31	3.64	27	3.17	168	19.76
Job security was directly affected	131	15.41	52	6.11	43	5.05	226	26.58
Full-time teleworking	222	26.11	212	24.94	78	9.17	512	60.23
Felt frustration regarding teleworking	83	9.76	28	3.29	32	3.76	143	16.82

**Table 2:** Results of data validity and reliability regarding collected data.

Construct	No. of Items	α ^1^ > 0.7	KMO ^2^ > 0.7	χ^2^; df; p ^3^	Eigen-Value	% Variance
Internal marketing	12	0.951	0.957	7750.50; 66; **	7.428	61.89
Job satisfaction	10	0.878	0.925	3547.14; 45; **	4.460	44.60
Job performance	5	0.803	0.821	1234.94; 10; **	2.279	45.58
	5	0.815	0.798	1397.33; 10; **	2.364	47.28

^1^—Cronbach α coefficient (to verify data reliability); ^2^—Kaiser-Meyer-Ohlin criterion (exploratory factor analysis) for every dimension; ^3^—Bartlett test of sphericity (χ^2^—Chi-square, df: degree of freedom, p: probability; ** *p* < 0.001).

**Table 3:** Operationalisation of constructs.

Construct	Measurement	Loading	EV/% of Var.
Internal marketing [56]	…teaches employees “why to perform tasks” and not just “how to perform them”.	0.814	12.986
	…offers employees not just training, but lifelong learning.	0.811	
	…developing employees’ abilities and know-how is a continuous process.	0.805	37.10%
	…communicates efficiently the vision to the employees.	0.796	
	…considers developing employee abilities and know-how an investment, not an expense.	0.784	
	…prepares employees to perform their tasks correctly.	0.781	
	…stresses the importance of employee communication.	0.780	
	…offers employees a vision in which they can believe.	0.777	
	…employees are trained to perform their duties correctly.	0.772	
	…is flexible in accommodating the various needs of employees.	0.769	
	…considers the needs and desires of the employees regarding the improvement of the working environment.	0.768	
	…communicates to the employees the importance of their key-role in task performance.	0.758	
Job satisfaction [119]	…I feel good at my workplace.	0.753	1.164
	…I like working for this company.	0.675	
	…I feel close to my co-workers.		
	…all my talents and abilities are put to good use at my workplace.	0.642	3.32%
	… my income is good.	0.617	
	…I get along well with my supervisors/chain of command.	0.537	
	…I believe organizational management is concerned about me.	0.534	
	…I feel safe regarding my workplace.	0.477	
	…I feel close to my co-workers.	0.446	
	…I believe work is good for my physical health.	0.427	
	…I am acknowledged when I perform my work/tasks well.	0.395	
Task performance [98]	…my task planning has always been good.	0.776	2.539
	…I plan my work to finish it on time.	0.705	
	…I always manage to separate primary tasks from secondary ones.	0.690	7.25%
	…I always think about the results I must obtain.	0.600	
	…I manage to perform my tasks well with minimal time and effort.	0.589	
Counterproductive work behavior [98]	I often complained to my co-workers regarding unimportant problems encountered at work.	0.764	1.891
	I talked to co-workers about the negative aspects of my work.	0.726	5.40%
	I focused on the negative aspects of a work situation, instead of on the positive aspects.	0.693	
	Work-related issues seemed more daunting than they were.	0.613	
	I talked to people from outside the organization about the negative aspects of my work.	0.609	

Notes: EV: Eigenvariance; % of var: percentage of variance; Factors in the order of their extraction. Extraction Method: Principal Axis Factoring. Rotation Method: Oblimin with Kaiser Normalization; Rotation converged in 9 iterations.

**Table 4:** The influence of internal marketing on job satisfaction and job performance.

Effects	Results
H1: Internal marketing → Job satisfaction	0.721 **
H2: Internal marketing →Task performance	0.030 ^n.s.^
H3: Internal marketing → Counterproductive work behavior	−0.041 ^n.s.^
H4: Job satisfaction → Task performance	0.296 **
H5: Job satisfaction → Counterproductive work behavior	−0.275 **

Note: ^n.s.^—not significant; ** *p* < 0.001; goodness-of-fit indices of the structural model: χ^2^/df: 4.665; GFI: 0.997; AGFI: 0.973; NFI: 0.995; CFI: 0.996; TLI: 0.974; SRMR: 0.0208 ≤ 0.08; RMSEA: 0.066 ≤ 0.08.

## Data Availability

The data presented in this study are available on request from the corresponding author. The data are not publicly available due the fact that they were obtained within the project POCU 123793 entitled “Researcher, future entrepreneur—New Generation of the Operational Program Human Capital 2014–2020.
